# Knowledge and utilization of family planning methods among people living with HIV in Kathmandu, Nepal

**DOI:** 10.1186/s12913-018-3643-3

**Published:** 2018-11-06

**Authors:** Rajani Pokharel, Geeta Bhattarai, Namuna Shrestha, Sharad Onta

**Affiliations:** 1 Department of Community Medicine and Public Health, Institute of Medicine, Tribhuvan University, Kathmandu, Nepal; 2 School of Public Health and Community Medicine, B.P. Koirala Institute of Health Sciences, Ghopa, Dharan, Nepal; 3 School of Public Health and Department of Community Medicine, Chitwan Medical College, Bharatpur, Nepal

**Keywords:** Family planning methods, HIV infected men and women, Utilization, Knowledge, Contraception, Fertility desire

## Abstract

**Background:**

Addressing family planning (FP) needs of people living with HIV is an effective response to HIV prevention. Healthy timing and spacing of births help ensure the health and well-being of all women and infants, regardless of their HIV-infection. In addition, preventing unintended pregnancies is an important strategy for prevention of mother to child transmission of HIV. The main purpose of this study was to explore the knowledge of family planning methods and identify factors affecting the utilization of such methods among HIV infected men and women attending two Anti-Retroviral Therapy (ART) sites of Kathmandu, Nepal.

**Methods:**

This was a descriptive cross-sectional study. Two ART sites of Kathmandu were purposively chosen and a sample of 265 respondents (both men and women) was selected based on proportionate probability random sampling. A structured questionnaire was administered face to face to all of the eligible participants.

**Results:**

The mean ± SD age of the participants was 36.62 ± 7.58 years. Sixty five percent of the respondents’ spouses were HIV positive. A majority of the respondents (72%) had heard of seven modern family planning methods. Considerably high number (72%) of the respondents or their spouses were using at least one of the method of family planning. The most common method was condom (83%) and the least common was IUCD (0.50%). The use of short acting (pills, depo-provera) and long acting (implant, IUCD) family planning methods other than condom dropped from 56.6 to 2% after diagnosis. Utilization of family planning methods was significantly associated with gender, education and HIV status of spouse. Males (Adjusted Odds Ratio (AOR) = 2.48, 95% CI = 1.20–5.07, *p* = 0.01) educated respondents (AOR = 3.27, 95% CI = 1.41–7.54, p = < 0.01) and individuals whose spouse were not infected with HIV (AOR = 4.70, 95% CI = 1.41–15.67, p = 0.01) were more likely to use FP methods.

**Conclusion:**

The tendency for HIV infected men and women to avoid additional child bearing in Nepal is higher compared to sub-Saharan Africa. However, the use of effective methods of family planning is low. Therefore, more effective counselling sessions by service providers regarding the availability and use of alternative family planning methods besides condom is necessary.

## Background

Globally, 35.3 million people are living with HIV infection, out of which 46% are women [1]. The ready availability and easy accessibility of antiretroviral therapies have however, considerably reduced the number of infections in recent years. The latest global data suggested that there were only 2.3 million new infections in 2012, which was 34% less than that in the year 2000 [1]. In 2015, the number of individuals with HIV infection in Nepal was estimated to be close to 40,000 with an overall national prevalence of 0.2% [2]. The HIV epidemic is concentrated among the people who injects drugs (PWID), men who have sex with men (MSM), transgender people (TG), male sex workers (MSW), female sex workers (FSW), the clients of sex workers, male labor migrants (MLM) and their wives. Heterosexual transmission is the most common mode of transmission for the HIV, accounting for 80% of new infections [3]. Five percent of the total infection is among children under 14 years. Within this group, mother to child transmission (MTCT) is the predominant mode of infection. Among the adult population, 65% of infections have occurred among males; and 35% of infections are in females, out of which around 26% are in the reproductive age group of 15–49 [3]. According to the national data of 2014, the annual number of new HIV positive pregnant women was 498 and the number of new infections among children was 178 [2].

Given a considerable proportion of PLHIV being in the reproductive age, the knowledge and use of family planning methods is of utmost importance in order to reduce the spread of infection to infants born to infected mothers. The appropriate timing and spacing of birth is important to all women regardless of the HIV-status in order to enhance their and their infants’ better health and well-being. Similar to uninfected women, women living with HIV have rights to determine the number and timing of their pregnancies and achieve their safe reproductive intentions. PLHIV can safely and effectively use all of the currently available contraceptive methods [4]. They should be informed about commonly available contraceptive options including emergency contraception. Female sterilization and vasectomy should be accessible to couples living with HIV who are interested in permanent sterilization [5].

Preventing unintended pregnancies among women living with HIV and AIDS is one of the strategies of the four-Pronged UN Strategy for Prevention of Mother to Child Transmission (PMTCT) of HIV [6]. The strategy focuses on good access to reproductive health services for PLHIV. A regular counseling and provision of contraception in a woman infected with HIV can ensure that unplanned pregnancy does not occur [4]. Planned pregnancies substantially reduces the risk of Mother to Child Transmission as couples that want to have children can wait until the HIV-positive mother’s viral load is low and she is healthy. Avoiding unplanned pregnancies reduces the number of children exposed to HIV in utero stage. Initiatives to expand the coverage and improve the quality of FP services should include efforts to address the FP needs of PLHIV. The strategy also aids in expanding the coverage and quality of FP services among this underserved population who are at high risk of unplanned pregnancy [5].

Most of the research related to the needs, knowledge and utilization of family planning methods in PLHIV has been concentrated in African nations [7–14] with very few in Asian countries [15–19]. In Nepal the available data on the uptake of family planning methods has not been disaggregated by HIV status. HIV related stigma and discrimination are still prevalent in both rural and urban areas that may contribute to the non-utilization of available services including family planning [20]. To date, little research has focused on identifying specific family planning needs and exploring the barriers for utilization of family planning services of Nepalese people living with HIV and AIDS.

The current study attempts to focus on the status of the use of FP methods and explore factors affecting the utilization among people living with HIV and AIDS in Kathmandu, the capital city of Nepal. This research finding is expected to contribute in enhancing the present understanding of family planning needs of people living with HIV and AIDS. It may also aid in developing better designed, better directed and more culturally sensitive localized intervention programs to optimize the utilization of family planning services by HIV infected men and women.

## Materials and methods

### Study sites

The study area included the following ART sites of Kathmandu valley:

- Tribhuvan University Teaching Hospital (TUTH), Maharajgunj (**Site 1**)
- Sukra Raj Tropical and Infections Disease Control Hospital, Teku (**Site 2**)

These ART sites were chosen because they cater services to the majority of people living with HIV and AIDS in and around Kathmandu valley.

### Study population

Currently married HIV infected women and men attending the above mentioned ART sites were included in the study. All of the participants were in the age range 15–49 years. Seriously ill individual who were unable to respond to the questionnaire were not enrolled.

### Sample size of study

The required sample size of 265 was determined as follows:

$$ \mathrm{n}={\mathrm{z}}^2\times \mathrm{pq}/{\mathrm{d}}^2={(1.96)}^2\times 0.5\times 0.5/{(0.05)}^2=384 $$

Where,

n = required sample size

z = 1.96 (95% confidence level)

p = prevalence of family planning method use among PLHIV = 0.5

d = precision or error allowed in the study = 0.05

q = (1-p)

While correcting for finite population

$$ \mathrm{New}\ \mathrm{Sample}\ \mathrm{size}=\frac{{\mathrm{z}}^2\times \mathrm{p}\ast \mathrm{q}}{{\mathrm{d}}^2+{\mathrm{z}}^2\mathrm{p}\ast \mathrm{q}/\mathrm{N}}+5\%\left(\mathrm{non}\hbox{-} \mathrm{response}\ \mathrm{rate}\right)=252+13=265 $$

Where *N* = 732, currently married PLHIV in pre- ART and ART in the study sites.

### Sampling

The two ART sites were purposively chosen for the study as they were sites with highest PLHIV registered. Selection of participants (men and women) in the age range 15–49 years at each of the sites was carried out using proportionate probability sampling. Our calculation required 13% (34) of the cases be included from site 1 and 87% (231) from site 2. Participants at each site were randomly selected based on the ART register maintained at respective sites. Fifty two percent of the respondents were male.

### Data collection, processing and analysis

A questionnaire tool was designed accordingly to meet the objectives of this study. Before its development, an in-depth search of related articles and relevant materials was conducted. A questionnaire was developed via adopting relevant questions from various sources. To ensure the reliability of the questionnaire, it was translated into Nepali and back translated into English. The Nepalese version of the tool was implemented for the study. The pretesting of the tool was conducted at the ART facility of Bir Hospital, a tertiary level government hospital in Kathmandu.

Face to face interviews were conducted using a structured questionnaire at each ART sites. Every completed questionnaire was checked at the end of the day to ensure its consistency and completeness. Coding of all of the variables was done to facilitate the data entry process.

The data was analyzed using SPSS V.16. Categorical variables were presented either as numbers or percentages. Means and standard deviations were presented for continuous variables. All independent variables were subjected to bivariate analysis. Association between independent variables and the utilization of family planning methods (dependent variable) were assessed using the chi-squared test and bivariate logistic regression. Those variables significantly associated in the bivariate analysis (*p* < 0.05) were further subjected into multivariate analysis in order to isolate factors independently associated with the utilization of family planning method.

### Variables

The variables in this study were divided into 7 categories. They were – a) Socio- demographic – this category included variables such as age, sex, age at marriage, education, religion and ethnicity b) HIV related - this category involved variables such as time since the diagnosis of the disease, use of ART, time since use of ART, spouse’s HIV status, disclosure with spouse and latest CD4 count c) Reproductive history and fertility desire – this category involved variables such as, the number of children of the participant, use of family planning before HIV diagnosis, HIV status of the youngest child, fertility desire, sexual intercourse in the last 6 months, and intention to get pregnant in the next 6 months d) Knowledge on family planning methods, this category constituted yes/no questions regarding knowledge on 7 commonly used family planning methods. e) Utilization of the family planning methods, this category consisted of variables such as, current use of FP methods, and utilization of emergency contraceptive in the past month. f) Perceived reasons for not using FP methods.

### Ethics

The Institutional review board of the Institute of Medicine, Tribhuvan University, Nepal approved this study. Written consent from respective health facilities was obtained prior to the enrollment of the participants. Oral informed consent was obtained from all of the participants before the interview. Participation in the study was voluntary. Participants were clearly informed about their rights to decline or withdraw from the study at any time, if desired. Confidentiality of the information collected during the study was assured.

## Results

Of the 265 participants enrolled in the study, 252 (95%) completed the questionnaire and were included in the analysis.

### Socio-demographic characteristics of the participants

The socio-demographic characteristics of the enrolled participants are presented in Table 1. There was a slight predominance of males (52%) in our study. The mean age of the participants was 36.62 ± 7.58 years. Most of the participants (86%) were above 30 years of age. A considerable proportion (34%) of the participants was less than 20 years of age at the time of their marriage. Eighty-three percent of the participants had obtained some form of education (literate) and 83% followed Hindu religion. Approximately half of the participants belonged to the Janajatis ethnic group.

**Table 1:** Age, sex, education and other relevant socio-demographic characteristics of participants enrolled in this study

Characteristics	Male Frequency in numbers	Female Frequency in numbers	Total Frequency in numbers (%)
	*N* = 131	*N* = 121	*N* = 252
Sex (*N* = 252)			
Male			131 (52)
Female			121 (48)
Age (*N* = 252)			
20–29	50	41	35 (14)
30–39	59	67	126 (50)
40–49	22	13	91 (36)
Age at marriage (*N* = 252)			
< 20	19	66	85 (34)
20–24	34	39	73 (29)
25–29	53	15	68 (27)
30+	25	1	26 (10)
Education (*N* = 252)			
Illiterate	22	21	43 (17)
Literate	109	100	209 (83)
Spouse Education (*N* = 252)			
Illiterate	19	16	35 (14)
Literate	112	105	217 (86)
Religion (*N* = 252)			
Hindu	120	87	210 (83)
Buddhist	8	18	26 (10)
Others^a^	3	16	16 (7)
Ethnicity (*N* = 252)			
Brahmin	30	46	76 (30)
Chhetri	34	11	45 (18)
Janjati	54	60	114 (45)
Dalit	13	4	17 (7)

^a^ Muslim and Christian

The findings of HIV infection related variables relevant to the objectives of this study are presented in Table 2. Slightly over half of the participants (54%) were diagnosed as having HIV within the last 24 months. A majority of the participants (84%) were receiving ART and the rest were in the process of receiving pre-ART. Sixty five percent of the participants responded that their spouses were also positive for the HIV infection. Eleven percent of participants responded that their spouse’s infection status was unknown, as they had not been tested. A considerably large proportion of the participants (86%) responded that they have disclosed their HIV status to their spouse. Fifty-five percent of the participants had CD4 count greater than 350 cells/mm^3^.

**Table 2:** HIV infection related characteristics of the participants

Variables	Male Frequency in numbers	Female Frequency in numbers	Total Frequency in numbers (%)
	*N* = 131	*N* = 121	*N* = 252
Time since HIV diagnosis			
Less than 24 months	60	76	136 (54)
More than 24 months	71	45	116 (46)
Initiated ART			
Yes	24	17	211 (84)
No	107	104	41 (16)
Time since ART initiation			
Less than 24 months	59	60	119 (56.4)
More than 24 months	49	43	92 (43.6)
Spouse’s HIV status			
Positive	60	103	163 (65)
Negative	50	11	61 (24)
Not tested	21	7	28 (11)
Disclosure with spouse			
Yes	23	12	217 (86)
No	108	109	35 (14)
Latest CD4 count			
< 350	67	45	112 (44)
> 350	64	76	140 (56)

### Reproductive history, sexual behavior, fertility desire and intention to get pregnant

Table 3 presents findings related to the reproductive history, sexual behavior, fertility desire and intention to get pregnant of the respondents. Eighty seven percent of the participants had at least one child. Forty two percent of the participants had two children. Among the participants with children, the HIV status of the youngest child was negative in 79.54%. 84.5% of respondents had had sexual intercourse in the past 6 months. Majority of the respondents (80%) had no further fertility desire, a psychological state in which someone has the personal motivation to have a child. Such motivation represents the first step towards a decision to act, typically followed by an intention to do so. Seventy eight percent of the respondents who had no child had desire for children whereas only 15 % of the respondents who had one to two children desired more. Of the respondents who indicated they would like to have children in the future, a separate question was asked if the respondents plan to initiate pregnancy in next 6 months. Among the respondents who had fertility desire and were sexually active in the past 6 months, only 6% had an intention to get pregnant in the next 6 months.

**Table 3:** Reproductive history, fertility desire and intention to initiate pregnancy

Variables	Male Frequency in numbers	Female Frequency in numbers	Total Frequency in numbers (%)
	*N* = 131	*N* = 121	*N* = 252
Number of children			
0	20	12	32 (13)
1	39	33	72 (29)
2	49	58	107 (42)
≥ 3	20	21	41 (16)
HIV status of youngest children (*N* = 220)			
Positive	10	10	20 (8)
Negative	90	85	175 (80)
Not tested	11	14	25 (12)
Fertility desire			
Yes	103	98	51 (20)
No	28	23	201 (80)
Sexual intercourse in the last 6 months			
Yes	129	84	213 (84.50)
No	2	37	39 (15.50)
Intention of childbearing in coming 6 months (*N* = 213)^a^			
Yes	117	83	13 (6)
No	11	2	200 (94)

^a^ Only sexually active respondents included

### Knowledge on family planning methods (heard of family planning methods)

Table 4 presents the knowledge of the respondents on seven modern family planning methods. The interviewer called a list of seven modern methods of family planning and the respondents were asked whether they had heard any of them.

**Table 4:** Knowledge on family planning methods

Knowledge on FP methods	Male Frequency in numbers	Female Frequency in numbers	Total Frequency in numbers (%)
Heard of condom			
No	1	0	
Yes	130	121	251 (99)
Heard of depo provera			
No	7	2	
Yes	124	119	243 (96)
Heard of pills			
No	15	0	
Yes	116	121	237 (94)
Heard of male sterilization			
No	12	16	
Yes	119	116	235 (93)
Heard of female sterilization			
No	14	6	
Yes	117	115	232 (92)
Heard of IUCD			
No	25	12	
Yes	106	109	215 (85)
Heard of implant			
No	39	16	
Yes	92	105	197 (78)

Responses were prompted

Knowledge on modern methods of family planning was almost universal, with 99 % of the respondents knowing at least one method of family planning. The most well-known method of family planning was condom (99%), followed by depo-provera (96%), pills (94%), male sterilization (93%), female sterilization (92%) and IUCD (85%). Implant was the least heard of FP methods among the respondents with 78% of the respondents having heard of this method.

### Utilization of the family planning methods

Before proceeding to questions on utilization on family planning methods, respondents were asked if they or their spouse were pregnant or in post-partum period during the interview. None of the respondents had positive response on this. Sixty eight percent of the respondents had used at least one method of family planning in the 12 months prior to diagnosis (Table 5). The use of FP methods includes the use either by the respondent or their spouse. The male respondents could report the use of female family planning methods used by their spouse such as implants, pills, IUCD, depo-provera, female sterilization; similarly, female respondents could report the use of male family planning methods used by their spouse such as male condoms and male sterilization. Among them, depo-provera (33%) was the most commonly used method followed by condom (27%) and pills (16%). A question was asked to those who reported the use of condom whether they have used the condom consistently or not in the past 3 months and almost all of them reported consistent use at least in the last 3 months.

**Table 5:** Utilization of family planning methods before and after HIV diagnosis

	Before diagnosis			After diagnosis		
	Male Response *N* (%)	Female Response *N*	Total response *N* (%)	Male Response *N*	Female Response *N*	Total Response *N* (%)
Utilization of FP methods			170 (67.46)			181 (71.82)
Methods used^a^						
Depo Provera	37	19	56 (32.94)	2	0	2 (1.10)
Male Condom	17	29	46 (27.05)	90	59	149 (82.32)
Pills	2	25	27 (15.88)			0
Male sterilization	9	6	15 (8.82)	9	6	15 (8.28)
Female sterilization	8	5	13 (7.65)	8	5	13 (7.18)
Implant	0	7	7 (4)			0
IUCD	2	5	7 (4)	0	1	1 (0.5)

^a^ FP method used either by respondent or spouse included

The use of family planning methods among the respondents increased slightly (71.8%) after the diagnosis. Among this group, the percentage used condoms increased sharply to 83%, whereas 17% used another method (male permanent method, female permanent method, depo-provera, IUCD). The use of short-acting (pills, depoprovera), long-acting (IUCD, implant) family planning methods other than condom dropped from 56.6 to 2% after diagnosis. Emergency contraceptive was utilized by 3% of the respondents in the past month.

Before diagnosis, 35% of respondents or their spouses using contraception relied on male methods (male condoms, male sterilization) whereas 65% used female methods (pills, depo-provera, IUCD, implant, female sterilization). After diagnosis There was a dramatic switch to male methods (91%) versus female methods (only 9%) after diagnosis.

Of those who did not want more children, 11% abstained from sex in the last 6 months, 72% were using family planning methods and 17% did not use any family planning methods.

### Perceived reasons for no-use of family planning methods

28.2% of the participants were not using any method of family planning. Among them, the most common reason (48%) for not using any family planning method was due to irregular sexual activity in the past 6 months. Other reasons were - both partners being HIV positive (18%), fear of side effects of using family planning methods (13%), spouse’s denial (10%) and no knowledge of appropriate FP for PLHIV (11%).

### Factors associated with the utilization of family planning methods

Bivariate analyses showed that sex, caste, educational status of the respondents, spouses’ education and HIV status of the spouse were significantly associated with the current utilization of family planning methods. A significantly higher proportion of participants in the age group 20–29, 30–39 years used FP methods compared to the other two groups. There was no association between FP utilization and age at marriage, religion, ethnicity, time of HIV diagnosis, use of ART, time since ART initiation, disclosure with spouse, latest CD4 count, HIV status of youngest child, fertility desire, ever having children and intention of child bearing (Table 6). On further multivariate analyses, three variables remained significant with the dependent variable. Modern contraceptive use was higher among men than women (AOR = 2.48 95% CI = 1.20–5.07, *p* = 0.01), those with a higher level of education (AOR = 3.27 95% CI = 1.41–7.54, *p* = < 0.01), and those who knew the HIV status of the spouse (AOR = 4.70,95% CI = 1.41–15.67, *p* = 0.01).

**Table 6:** Factors associated with current utilization of family planning

Characteristics	Current Utilization of Family Planning Methods		OR (95%CI)	*p*-value	aOR (95% CI)	*p*-value
	No (n)	Yes (y)				
Age						
20–29	6	29	**2.88 (1.08–7.65)**	**0.03**	2.47 (0.80–7.76)	0.12
30–39	31	95	1.57 (0.59–4.1)	0.35	0.97 (0.32–2.87)	0.96
40–49	34	57	1			
Age at Marriage						
< 20	28	57	1	0.73		
20–24	22	51	1.13 (0.58–2.23)	0.15		
25–29	15	53	1.73 (0.83–3.60)	0.47		
30+	6	20	1.63 (0.59–4.53)			
Sex						
Female	47	74				
Male	24	107	**2.83 (1.59–5.02)**	**< 0.05**	**2.48 (1.20–5.07)**	**0.01**
Education						
Illiterate	22	21				
Literate	49	160	**3.42 (1.74–6.74)**	**< 0.05**	**3.27 (1.41–7.54)**	**0.005**
Spouse Education						
Illiterate	15	20	**2.15 (1.03–4.49)**	**0.04**	0.85 (0.32–2.30)	0.75
Literate	56	161				
Ethnicity						
Brahmin	34	42	1		1	
Chhetri	8	37	**3.77 (1.54–9.09)**	**< 0.01**	2.40 (0.59–9.71)	0.21
Janjati	24	90	**3.03 (1.60–5.74)**	**< 0.01**	0.49 (0.11–2.13)	0.34
Dalit	5	12	1.94 (0.62–6.05)	0.28	0.82 (0.21–3.22)	0.78
Religion						
Hindu	51	156	1			
Buddhist	11	15	0.44 (0.19–1.03)	0.06		
Others	9	10	**0.36 (0.14–0.94)**	**0.05**		
Time since HIV Diagnosis						
Less than 24 Months	41	95				
More than 24 Months	30	86	1.23 (0.71–2.15)	0.48		
Use of ART						
No	13	28	0.81 (0.39–1.68)	0.58		
Yes	58	153				
Time since ART initiation (*N* = 211)						
Less than 24 months	30	89	1.23 (0.66–2.26)	0.50		
More than 24 months	27	65				
Spouse’s HIV status						
Positive	54	109	1			
Negative	7	54	**3.82 (1.63–8.96)**	**0.002**	**4.70 (1.41–15.67)**	**0.01**
Not tested	10	18	**4.28 (1.42–12.9)**	**0.006**	2.22 (0.83–5.95)	0.11
Disclosure with spouse						
No	7	28	1.67 (0.69–4.02)	0.25		
Yes	64	153				
Latest CD4 count						
Less than 350	29	83	1.22 (0.70–2.13)	0.47		
More than 350	42	98				
No of living children						
0	10	22	1			
1–2	50	124	1.44 (.52–3.96)	.47		
3+	11	35	1.28 (.60–2.72)	.51		
HIV status of youngest children (N = 220)						
Positive	7	13	1			
Negative	45	130	1.55 (0.58–4.14)	0.37		
Not tested	6	19	0.58 (0.16–2.14)	0.42		
Fertility desire						
No	56	145	0.92 (0.47–1.82)	0.82		
Yes	15	36				
Intention of becoming pregnant in coming 6 months						
No	33	167	0.44 (0.13–1.52)	0.24		
Yes	4	9				

Significant values are highlighted in boldface

## Discussion

The aim of the study was to assess knowledge on family planning methods and explore factors affecting the utilization of family planning methods among a sample of married Care & Treatment clients in Kathmandu.

This study revealed that a considerable proportion of participants had heard of at least one method of family planning. Similar finding was shown by a study conducted in Northern Uganda that reported that there was a high level of knowledge on family planning methods among the PLHIV surveyed (96%) [8]. Supporting this finding is another cross-sectional descriptive study conducted in the year 2013 in Cameroon. This study found that 98% of the women participants knew at least one method of contraception [10]. In contrast, a study conducted among people living with HIV and AIDS attending clinic in Nigeria reported that only 7% of the respondents had good knowledge of family planning methods [21].

In this study, more respondents had heard of the condom than any other method. This finding is in line with a study that was conducted in Kaski district of Nepal in a similar population where almost all of the participants enrolled had heard of condom [15].

The current use of the modern methods of family planning was found to be higher than that of the general population (68% versus 43%) [22]. It should be noted that the Nepal Demographic Health Survey (NDHS) measured the Contraceptive Prevalence Rate (CPR) by asking women respondents only whereas in our study we included the responses from both men and women. The rate of use was similar to that of the study done in Kaski, Nepal (70%) [9, 15]. The rate was substantially lower than the rate of FP use among HIV infected men and women in India (92%) [23]. However, the rate was considerably higher than some other African nations [19, 24]. The relatively high rate of use of FP methods found in our study is due to the high use of condom. When condoms were excluded the current use of modern methods dropped to 17%. Hence, this finding may need to be cautiously interpreted as clients infected with HIV can over report the use of condom [25]. If so, the actual utilization for family planning methods may be grossly overestimated.

The comparison of the use of family planning methods before and after HIV diagnosis suggest remarkable decline in the use of non-condom contraceptives. This finding is in line with the studies in India, Uganda, Iran and Swaziland [19, 26–29] that reported decline in use of contraceptive methods other than condom post HIV diagnosis. Studies from India have argued the lack of discussion about non-condom contraceptives by health care providers as one of the barriers for use of non-condom contraceptives [19, 26]. Fears of complications for pills and injectable and the cost for implants, intrauterine devices were major reasons for non-usage of these methods by PLHIV in Uganda [27]. A mixed method study done in Iran reported PLHIV were not willing to use methods other than condom because healthcare providers recommended the use of condoms as the main method of contraception [28]. Similarly study in Swaziland has found the fear of side effects and the programmatic focus on condoms as reasons for non-utilization of methods other than condom [29]. Given this, high dependency on male condoms it would be worthwhile to explore whether health care providers are making methods other than condoms available or not. It is well known that among all contraceptives, condom has the highest failure rate [30]. Despite this fact, most of them were using condom only. A very good contraceptive protection must be ensured to PLHIV if they do not want any pregnancy. Since women have a potential risk of pregnancy due to unprotected sex, women need to use effective methods that are women controlled along with condoms. This indicates need for effective counseling on different methods of family planning by health care providers by training and sensitizing them on the current consensus that HIV-infected women can use all available contraceptive methods to help them make informed choice.

The most common perceived reason for non-utilization of family planning methods among the participants in this study was irregular sexual activity in the past 6 months Other reasons such as fear of side effects of using FP methods, spouse refusal to use contraceptive, lack of awareness regarding proper FP method for infected individuals, both partners being HIV positive clearly indicate a lack of sufficient counseling and awareness regarding FP methods to the PLHIV group reflects the need for proper counseling on all methods of family planning for this group. A study conducted in a similar population in Kaski, Nepal found receiving counseling sessions on family planning significantly associated with the uptake of family planning methods [15]. The same study documented that lack of knowledge about the availability of FP services lack of resources to access and fear of stigma and discrimination as the major reasons for non-utilization of family planning methods. Factors such as fear of side effects, reduction in pleasure, misinformation, negative perceptions, gender-inequality, bad experiences with using some methods and health concerns have been identified as other reasons for non-utilization of family planning in similar studies done in Ghana [9] and Uganda [7].

Only 20 % of the participants desired children or more children and of that even lower percentage seek to become pregnant in coming 6 months. This finding was lower than the finding of such studies in other regions of the world especially Africa [23, 31–33] but higher than in India [16]. Cultural factors are also important for fertility desire especially in sub-Saharan Africa and Asia [33]. An ethnographic study among PLHIV in Nigeria showed the importance of marriage and parenthood in their life aspirations, regardless of their HIV status [34]. Similarly other studies from Africa reported societal pressure as reason for wanting more children [32, 33]. Although the Nepalese society also regards child bearing as an important life goals but HIV status seems to have negative impact on that. Even though we did not explore factors associated with fertility desire in our study, previous study has found that the knowledge of own HIV positive sero-status was significantly associated with limiting fertility desire and seek to prevent pregnancy using contraception [16, 35]. This could be true in our study as well, as all of the participants enrolled were aware of their HIV status. Similarly, the study from India has reported the number of children they already had as reasons for not wanting more children [16] which could also be true in our case. The total fertility rate (TFR) in Nepal is 2.17 and our study found that the mean number of children for the respondents as 1.96(±0.06) [22]. The fertility desire was lower among the respondents with one to two children compared to those with no children. Thus, respondents may want to curtail further childbearing for reasons other than their HIV status. Even so, proper counseling to these people for safe pregnancy and effective use of family planning methods is required in order to reduce mother to child transmission to zero. Future research that examines fertility desires among PLHIV should include cultural factors in the theoretical framework in order to provide a holistic understanding of the fertility desires of HIV infected men and women [34].

Fertility desire was not significantly associated with the use of family planning methods in our study. However, many studies have reported fertility desire as predictor of family planning use. Participants who had no fertility desire were more likely to use contraceptive (AOR: 3.11, 95% C.I: 1.46, 6.64) in a study done in Ethiopia [36] and participants whose spouse did not have desire for children were two times more likely to use contraceptive (AOR =2.19, 95% CI: 1.10–4.36; *p* = .025) in a study from Uganda [8]. Fertility desire was not a predictor of family planning use in our study. This finding is hard to explain but we assume that despite fertility desire, HIV infected men and women don’t have intention to initiate pregnancy and don’t involve in sexual intercourse because they are worried of mother to child transmission and hence have low use of family planning methods.

The current study found that the respondents’ sex, education status and, spouse’s HIV status were significantly associated with the use of contraception. Males were approximately three times more likely to use FP methods as compared to females. This might be due to a higher use of male condom that is encouraged by health workers to PLHIV for the prevention of sexual transmission of HIV. Many HIV infected individuals thus opt for condom for the prevention of sexual transmission as well as for family planning [8].

In this study, literate participants were also three times as likely to utilize FP methods as iliterate participants. This findings imply a significant role of education staus of participants in utilizing available health care services. Education status was reported as predictor of use of family planning methods by a few other studies as well [8, 12, 29, 37] Similarly, respondents whose spouse’s were negative were four times more likely to use FP methods than those whose spouse’s status was positive (Table 6). Higher utilization of FP services among participants whose spouse were tested negative for PLHIV as compared to those whose spouses were positive or were not tested for the status suggests that the HIV infected individuals were concerned about their spouses health and well-being. This finding is in line with with one another study conducted in Ghana where contraceptive use was strongly associated with partner knowledge of HIV status (AOR = 3.64; 95% CI 1.36–9.72; *p* = 0.01) [9].

The results of this study need to be considered in relation to its limitations. First, the consistency on the use of contraceptive methods by the participants both before and after HIV diagnosis was not confirmed in our study, which could have led to a higher reporting of contraception use. However, query about consistency of condom use in the past 3 months was asked and almost all of them reported consistent use at least in the last 3 months. Second, this study could have underestimated or overestimated the use of spouse contraception as we relied on the participant’s self-report of spouse’s use of contraceptives. Men may have underreported non-condom contraceptive use among their wives because they were not in direct control of their use or may not have been aware about their use. Third, this study did not explore the reasons behind the non-use of family planning methods among participants who responded no fertility desire. This objective was beyond the hypothesis of this study and needs to be explored separately. Nevertheless, this study has provided us with important information on the family planning practices and knowledge among people living with HIV in a city of a south Asian developing country where high risk population resides.

## Conclusion

HIV infected adults in Nepal avoid child bearing to a greater extent than those in the sub-Saharan Africa. A considerable number of participants reported no use of highly effective FP methods, i.e., methods other than condoms. The switch to condom use from other contraceptives was high post HIV diagnosis. Almost all of the participants had heard of condom as a family planning method and a majority was using it as a FP method. Given this high dependency on male condoms it would be worthwhile to explore whether health care providers are making methods other than condoms available or not. This implies that more emphasis needs to be given on counseling on the use of different FP methods by the health care providers among individuals with HIV in order to prevent mother to child transmission. The counseling sessions should also highlight on the benefits and safety of FP services. This will ensure that PLHIV are able to make informed decisions about their sexual and reproductive health and have access to appropriate methods.

## Abbreviations

ART: Anti Retroviral Therapy
FP: Family planning
IUCD: Intrauterine contraceptive device
OCP: Oral contraceptive pills
PLHIV: People living with human immunodeficiency virus
PMTCT: Prevention of mother to child transmission
